# Late diagnosed necrotizing fasciitis as a cause of multiorgan dysfunction syndrome: A case report

**DOI:** 10.1186/1757-1626-1-125

**Published:** 2008-08-23

**Authors:** Piotr Smuszkiewicz, Iwona Trojanowska, Hanna Tomczak

**Affiliations:** 1 Department of Anesthesiology, Intensive Therapy and Pain Treatment, University Hospital, Przybyszewskiego 49, 60-355 Poznan, Poland; 2 Central Laboratory of Microbiology, University Hospital, Przybyszewskiego 49, 60-355 Poznan, Poland

## Abstract

Necrotizing fasciitis is a rapidly progressive, life-threatening soft tissue bacterial infection. We present a serious case of a 43-year-old male who suffered from necrotizing fasciitis of the left leg in whom a delayed diagnosis caused multiorgan dysfunction.

Early recognition of important symptoms is essential in the management and surgical debridement of necrotizing fasciitis. Treatment should include comprehensive supportive measures (early goal-directed therapy, adequate ventilation strategy, activated protein C dosage, tight glucose control, steroids, renal replacement therapy) and early antibiotic therapy based on microbiologic monitoring. The pathophysiology and etiologic factors of necrotizing fasciitis are discussed.

## Introduction

Necrotizing fasciitis (NF) is a rapidly progressive, life-threatening soft tissue infection. It develops from a bacterial infection, most often with group A Streptococcus (GAS). However, mixed aerobic and anaerobic Gram positive (G+) and Gram negative (G-) flora may also be the infectious agents. Their growth takes place in an environment of local tissue hypoxia with decreased function of polymorphonuclear leukocytes, particularly in patients with the following risk factors: medical compromise (e.g., systemic illnesses, immunosuppressive medications), trauma, recent surgery, recent birth, diabetes mellitus, vascular insufficiency, renal and hepatic failure, cancer, organ transplants [1]. The key to increasing the chances survival for a patient with NF is early and proper diagnosis.

## Case presentation

A 43-years-old Caucasian male (height 198 cm, weight 115 kg, tobacco smoker – 1 packet of cigarettes/day) was admitted to the intensive care unit (ICU) of a university hospital from a municipal hospital because of multiorgan dysfunction syndrome associated with infection and necrosis of the skin, subcutaneous tissue, and fascia of the left lower leg. The signs and symptoms of the disease – edema and pain affecting the left leg-began 7 days before admission. The patient's medical history was significant for hepatitis C (HCV), treated with ribavarin and interferon, and diabetes mellitus treated with insulin. On the day of admission the HCV-RNA test was negative. A mosquito bite was the probable triggering event.

**Figure 1:**
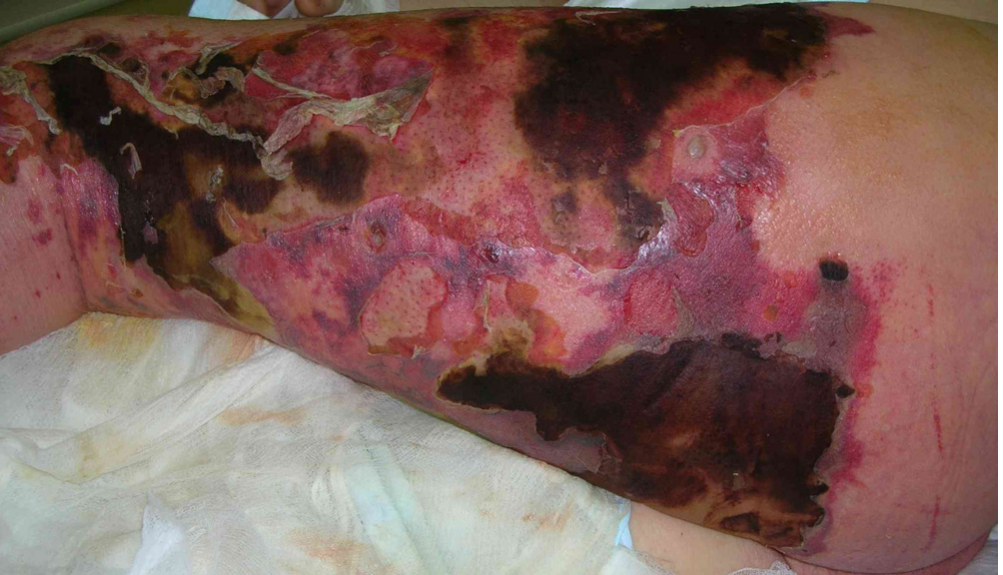
**43-year-old male.** Preoperative photograph on the day of admission. Extensive erythema and necrosis of the left leg.

On admission to the ICU the patient was confused, with a Glasgow Coma Scale (GCS) of 9. He was afebrile, had tachycardia, with a heart rate of 120 beats per minute, hypotension, with a blood pressure of 90/50 mmHg, and relative respiratory insufficiency (oxygen saturation of 88% – 90%). He had moderately-dilated pupils with a delayed light reflex. We noted crepitus over the bilateral lung fields, a tender abdomen with absent bowel sounds, and oliguria. There were numerous hemorrhagic extravasations on the skin. Examination of the left leg revealed diffuse edema of the skin, with marked erythema and necrosis; margins of infection were sharply demarcated, spreading onto the buttock and perineum on the left side (Figure 1). Laboratory tests showed a metabolic acidosis, with the following values: base excess -15 mmol/l, lactate level 7.3 mmol/l, PaO_2 _64 mmHg, platelet count 29,000/mm^3^, International Normalized Ratio (INR) 2.24, serum creatinine 691.6 μmol/l, bilirubin 62.6 μmol/l, and C-reactive Protein (CRP) 224.6 μg/dl.

The Acute Physiology and Chronic Health Evaluation (APACHE) II score on the day of admission was 31 points and the Sequential Organ Failure Assessment (SOFA) score was 18 points. The patient required fluid resuscitation, endotracheal intubation, mechanical ventilation in BiPAP (bilevel positive airway pressure) mode, continuous intravenous infusion of catecholamines (epinephrine, norepinephrine) and low doses of steroids to restore blood pressure. After obtaining cultures from the affected tissue, blood and bronchoalveolar lavage (BAL) in appropriate media, empirical, broad-spectrum antibiotics were immediately administered (meropenem 3 g/day in a 3-h infusion, vancomycin 3 g/day in continuous infusion, metronidazole 1.5/day in 3 divided doses). Because of progressive organ dysfunction, therapy was started with activated protein C, in doses of 24 μg/kg/h; continuous veno-venous hemofiltration (CVVH) was also started. After several hours, the patient developed atrial fibrillation with a ventricular rate of 140 beats per minute and his blood pressure decreased to 80/50 mmHg. Electrical cardioversion was performed three times, followed by continuous infusion of cordarone (12 mg/kg/day). During this time, intensive fluid resuscitation was administered. During the first day, the patient received 12,800 ml of fluid (colloids and crystalloids). His unstable condition did not allow for early surgical management.

**Figure 2:**
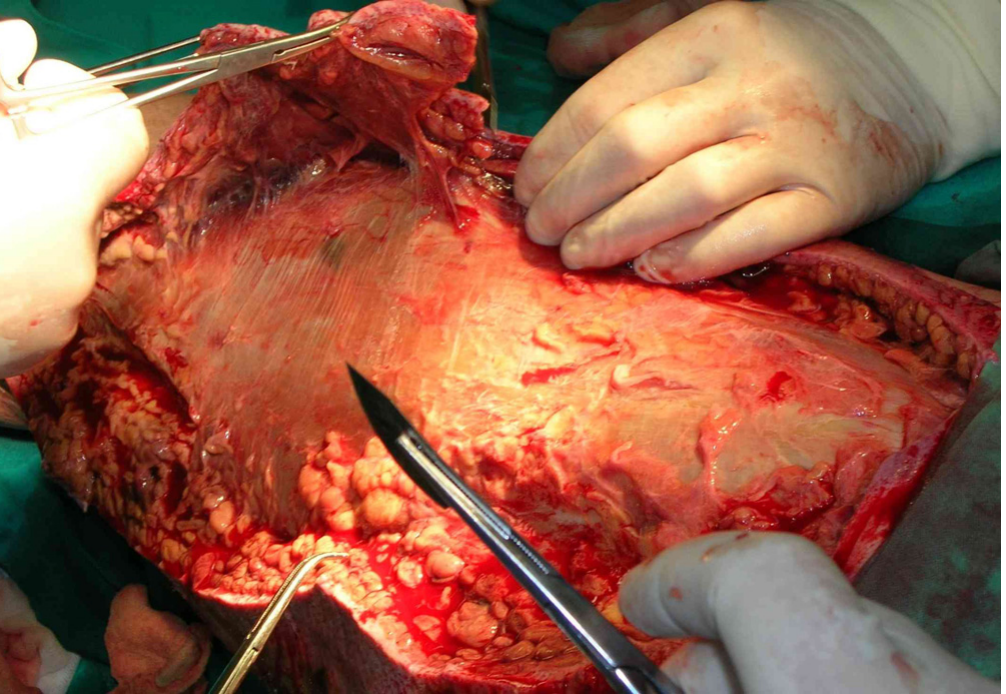
**43-year-old male.** Seventh day of treatment. Intraoperative photographs: necrotic tissue from the left leg was surgically debrided.

**Figure 3:**
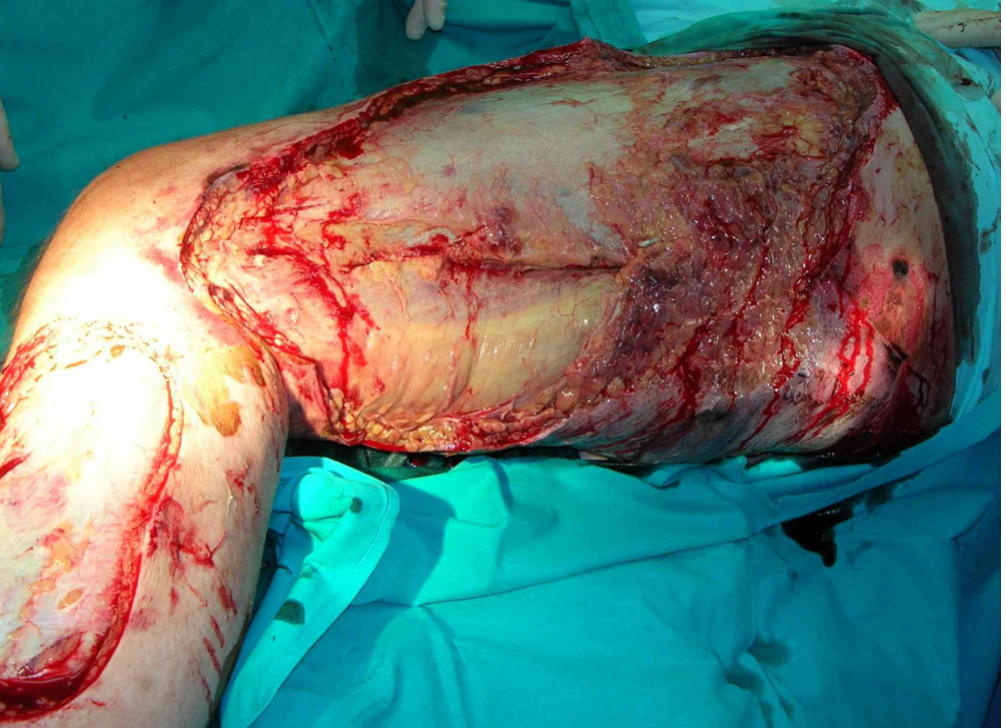
**43-year-old male.** Seventh day of treatment. Intraoperative photographs: necrotic tissue from the left leg was surgically debrided.

The cultures from the affected areas of skin contained Streptococcus pyogenes and Staphylococcus sciuri. Based on sensitivity results, vancomycin and metronidazole were discontinued, and penicillin G was started at a dose of 60 million units a day (intravenous) in 6 divided doses. Because the patient's poor condition persisted and the microbes were sensitive to clindamycin, it was added at 2.7 g/day in 3 divided doses. In spite of the lack of information about G-pathogens, meropenem was continued. The patient also received 40 g of commercially available gamma-globulin (intravenous), 8 units of packed red blood cells, 6 units of fresh frozen plasma, and 8 units of platelets. After several days his condition improved, with urine output of 100–150 ml/h and resolution of his metabolic acidosis. On the sixth day, the patient's hemodynamic parameters deteriorated again, with tachycardia to 160 beats per minute and a decrease in blood pressure to 85/60 mmHg. CVVH was administered again for detoxification. On the seventh day, the necrotic tissue was surgically excised (Figures 2, 3). We also noted a progressive decline in the white blood cell count to 2,200/mm^3^, and administered granulocyte colony stimulating factor.

Cultures of the BAL showed 10^4 ^colony-forming units/ml of methycillin resistant Staphylococcus aureus (MRSA). (The patient had been a carrier of methycillin susceptible Staphylococcus aureus [MSSA] isolated from his nose vestibule on admission.) Because MRSA also was present in blood cultures and the patient's condition remained poor, linezolid 1.2 g a day in 2 divided doses was initiated based on additional results of susceptibility tests. After 10 days of intensive treatment, the patient was hemodynamically stable, and we discontinued mechanical ventilation. 25 days after admission the patient was discharged to the trauma surgery ward for plastic reconstruction of his wounds.

## Discussion

NF is a relatively rare disease, in which infection and necrosis are most often located on the lower legs and perineum. In 1883, Fournier reported a case of fulminant gangrene of the testes in an otherwise healthy man. In 1952, Wilson [2] used the name "necrotizing fasciitis" to describe the same disease, located on a different part of the body. Mortality in NF ranges from 20 to 30%, but is considerably higher, approximately 80%, when the disease is associated with sepsis and renal failure [3,4].

The APACHE II score provides an estimated prediction of ICU mortality based on a number of laboratory values and signs, taking both acute and chronic diseases into account. The APACHE II and the SOFA score reflect the status of the organ systems and are widely used, mainly as indices of illness severity and for outcome prediction. In this patient, the APACHE II score reached a very high value (31 points) with a predicted mortality of 73%.

Early diagnosis is not always possible, because signs such as erythema, tenderness, swelling, and fever accompany other inflammatory states of skin and subcutaneous tissue (e.g., cellulitis). Wong [5] concludes that in 89 examined patients with NF, only approximately 15% were diagnosed properly. Delay in establishing the correct diagnosis may have catastrophic repercussions, such as dynamically developing organ dysfunction, particularly in patients, such as this, with risk factors. Cornia et al [6] emphasized that early diagnosis is extremely important for the implementation of appropriate empiric antibiotics and this must be based on a clinician's high index of suspicion. Advanced necrosis and organ dysfunction in our patient indicated that the proper diagnosis was seriously delayed (symptoms began 7 days before admission). According to Sendi [7] patients with NF can be classified into two categories. The first category consists of patients with antecedent surgery, diabetes mellitus and vascular diseases in whom the infection is usually polymicrobial. The second category consists of patients in whom NF is induced by a single pathogen, usually GAS, and these patients usually have no underlying diseases. Our patient had perturbations in the functioning of his immunological system. The predisposing factors were insulin-dependent diabetes and hepatitis C, treated with immunosuppressive drugs. The etiologic agent was GAS and staphylococcus species. GAS is classified depending on the presence of superficial proteins – T and M antigens. In the pathophysiology of NF, antigen M plays an especially significant role because it is responsible for the inefficiency of phagocytosis, which determines the intensity of the pathogenic virulence. Protein M diminishes activation of the alternative pathway in the complement system, by inhibiting the binding of the C3 component to the microbe cells and increasing their resistance to phagocytosis. The expansion of NF depends on the action of proteolytic toxins and enzymes, produced by bacteria, that are responsible for the rapid spread of infection, lead to necrosis of the tissues. The activation of cascade systems and endothelial injury disturb tissue perfusion, leading to death due to full-blown septic shock [8]. Pivotal in the treatment of NF is surgical excision of necrosis as soon as possible and widely accepted intensive therapy, which relies on properly selected antibiotics and supportive treatment for organ failure. Surgical procedures rely upon executing multiple, wide incisions, ensuring outflow of infected secretions and removal of necrotic tissue. In the patient presented here, radical surgery seemed to be dangerous due to serious shock and disseminated intravascular coagulation. The patient's body mass in combination with the necrosis affecting a large proportion of his body surface meant that a complete excision would carry the risk of massive bleeding. Moreover, we were forced to employ CVVH with heparin infusion and activated protein C. Thus, early surgical necrosis excision seems to be less dangerous in patients without shock and coagulation perturbation and after stabilization of vital parameters. Endorf et al [9] presented 65 cases of NF, in which all surgical procedures occurred over a mean of 5.3 days. In addition, Miller et al [10] reported that this time varied from 3 days to over 3 months. In spite of the fact that early surgical treatment was linked to increased mortality, rapid surgical intervention is still recommended [9,11].

The influence of streptococcal toxins and the subsequent physiological reactions suggest that penicillin G and clindamycin should be administered empirically. Because of our patient's prior stay in another hospital, we empirically initiated carbapenem and vancomycin, to cover nosocomial pathogens. Penicillin and clindamycin were administrated on the third and fourth days of treatment, respectively, after obtaining microbiological susceptibility tests. However, an increasing number of published reports have documented the inefficiency of penicillin in treating serious GAS infections [8]. The superior effects of clindamycin result from its inhibition of toxin and protein M synthesis, a potency that is independent of the size of the inoculum or GAS growth phase, and its longer lasting postantibiotic effect. There are reports about clindamycin's ability to modify the function of the immune system in GAS infection [8]. Thus, clindamycin remains a reliable, well-established and effective antibiotic in treating NF.

In this case, the patient presented with signs and symptoms of severe sepsis, for which intensive supportive care measures are the standard of care. These measures include the 6-hour resuscitation bundle, which consists of lactate level monitoring, early cultures and antibiotics, and early goal-directed therapy (intensive fluid resuscitation and administration of catecholamines). The standard intensive measures also include a 24-hour management bundle with a physiological approach to ventilation, administration of activated protein C (which has a multifaceted mechanism of action), tight glucose control, low-dose steroids and renal replacement therapy [12]. Alternative measures, such as hyperbaric oxygen, immunoglobulin or granulocyte colony-stimulating factor in granulocytopenic patients, may also be effective, but have not yet been tested in controlled studies [8].

Treatment of NF is exceedingly expensive. A study performed in the USA in 2001 showed that the entire cost of treating one patient amounted to approximately 150,000 US dollars [11].

## Conclusion

Although NF is a rare disease, physicians must be well trained in its diagnosis and treatment, to facilitate prompt interdisciplinary management. Clinicians assessing patients with rapidly progressive skin infections should be aware of the symptoms and have a high index of suspicion.

Patients with NF should be treated in ICUs that offer a wide range of supportive care for organ dysfunction, microbiological monitoring and surgical procedures. NF is one of the greatest challenges from both a therapeutic and financial perspective.

## Consent

Written informed consent was obtained from the patient for publication of this case report and accompanying images. A copy of the written consent is available for review by the Editor-in-Chief of this journal.

## Competing interests

The authors declare that they have no competing interests.

## Authors' contributions

PS and IT were involved in the treatment of the patient and wrote and finalized the manuscript. HT performed the microbiological examination and helped to draft the manuscript. All authors read and approved the final manuscript.
